# Supplementary material for: Small leucine-rich proteoglycans inhibit CNS regeneration by modifying the structural and mechanical properties of the lesion environment
Source: Nat Commun. 2023 Oct 26;14:6814. doi: 10.1038/s41467-023-42339-7 (PMC10603094; doi:10.1038/s41467-023-42339-7)
Supplement: Supplementary file 1 — Supplementary Information [file 41467_2023_42339_MOESM1_ESM.pdf]

Supplementary Information

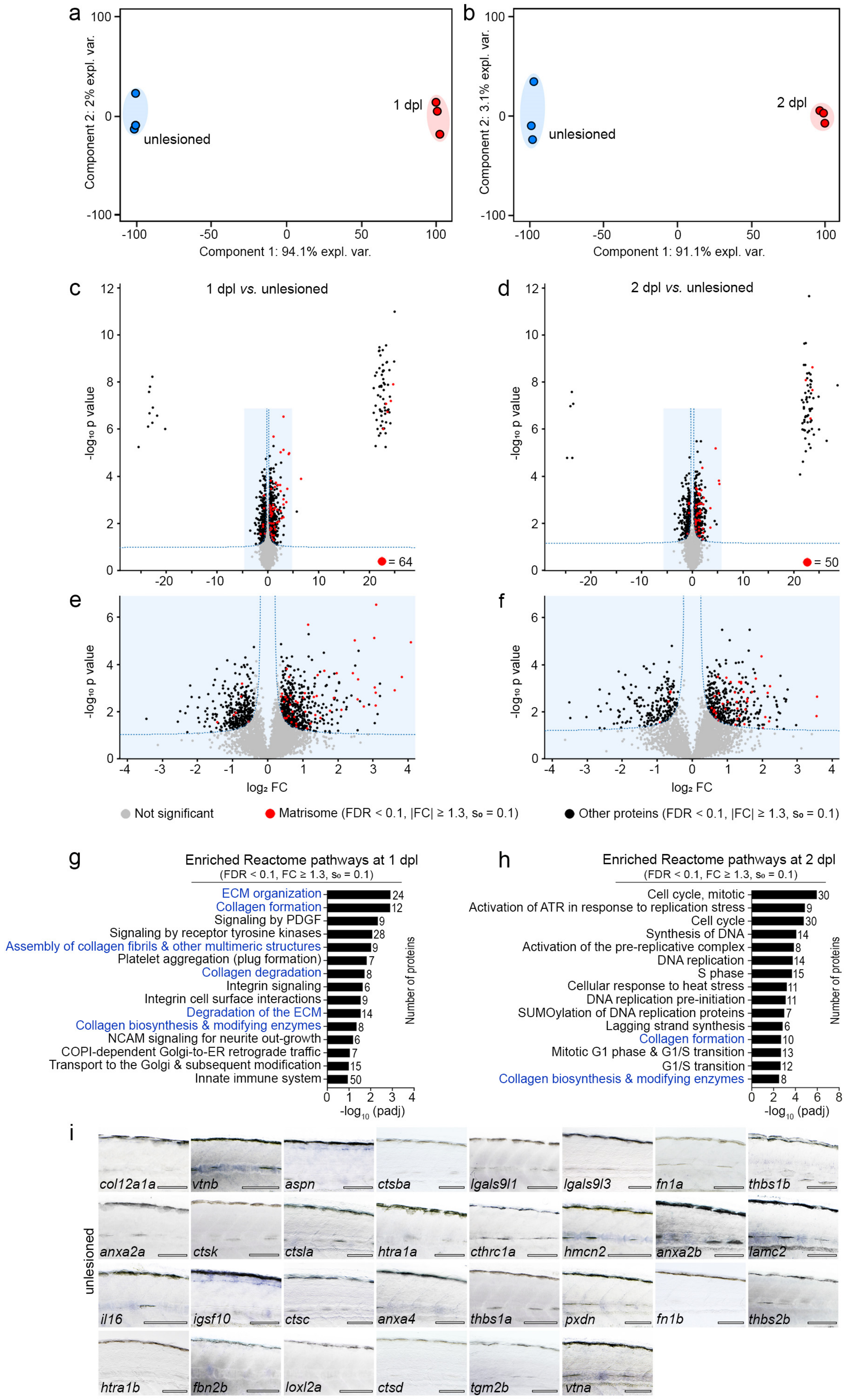
a
Component 2: 2% expl. var.
Component 1: 94.1% expl. var.
1 dpl
unlesioned
b
Component 2: 3.1% expl. var.
Component 1: 91.1% expl. var.
2 dpl
unlesioned
c
1 dpl vs. unlesioned
-log₁₀ p value
= 64
d
2 dpl vs. unlesioned
= 50
e
f
log₂ FC
Not significant
Matrisome (FDR < 0.1, |FC| ≥ 1.3, s₀ = 0.1)
Other proteins (FDR < 0.1, |FC| ≥ 1.3, s₀ = 0.1)
g
Enriched Reactome pathways at 1 dpl
(FDR < 0.1, FC ≥ 1.3, s₀ = 0.1)
ECM organization 24
Collagen formation 12
Signaling by PDGF 9
Signaling by receptor tyrosine kinases 28
Assembly of collagen fibrils & other multimeric structures 9
Platelet aggregation (plug formation) 7
Collagen degradation 8
Integrin signaling 6
Integrin cell surface interactions 9
Degradation of the ECM 14
Collagen biosynthesis & modifying enzymes 8
NCAM signaling for neurite out-growth 6
COPI-dependent Golgi-to-ER retrograde traffic 7
Transport to the Golgi & subsequent modification 15
Innate immune system 50
Number of proteins
-log10 (padj)
h
Enriched Reactome pathways at 2 dpl
(FDR < 0.1, FC ≥ 1.3, s₀ = 0.1)
Cell cycle, mitotic 30
Activation of ATR in response to replication stress 9
Cell cycle 30
Synthesis of DNA 14
Activation of the pre-replicative complex 8
DNA replication 14
S phase 15
Cellular response to heat stress 11
DNA replication pre-initiation 11
SUMOylation of DNA replication proteins 7
Lagging strand synthesis 6
Collagen formation 10
Mitotic G1 phase & G1/S transition 13
G1/S transition 12
Collagen biosynthesis & modifying enzymes 8
Number of proteins
-log10 (padj)
i
unlesioned
col12a1a
vtnb
aspn
ctsba
lgals9l1
lgals9l3
fn1a
thbs1b
anxa2a
ctsk
ctsla
htra1a
cthrc1a
hmcn2
anxa2b
lamc2
il16
igsf10
ctsc
anxa4
thbs1a
pxdn
fn1b
thbs2b
htra1b
fbn2b
loxl2a
ctsd
tgm2b
vtna

Supplementary Fig. 1 | Mass spectrometry-based quantitative proteomics reveals changes in ECM composition during zebrafish spinal cord regeneration.

a-b) Principle component analysis of mass spectrometry-based proteomics data using quantitative values of all identified proteins across samples. Lesioned samples (1 dpl, 2 dpl) cluster distinct from unlesioned age-matched control samples (4 dpf, 5 dpf). Each data point represents one independent biological replicate.

c-f) Volcano plots of all quantified proteins for the given analyses (1 dpl *vs*. unlesioned age-matched controls, (**c, e**); 2 dpl *vs.* unlesioned age-matched controls, (**d, f**)) with their log_2-transformed ratios of the mean-centered abundances (FC, fold change) and -log_{10}-transformed *P*-values (two-tailed t-test). Dashed lines indicate the threshold of a permutation-based FDR correction for multiple hypotheses ($FDR < 0.1$, $s_0 = 0.1$) for identification of significantly altered abundances. Proteins with significantly altered abundance were further filtered by $|FC| \geq 1.3$. Area highlighted (blue) in (**c**) and (**d**) is shown at a different scale in (**e**) and (**f**).

g-h) Reactome pathway analysis of differentially enriched proteins reveals ECM-associated terms (blue) being overrepresented at 1 dpl (**g**) and 2 dpl (**h**). Bonferroni correction.

i) Expression of the indicated genes in the trunk of 4 dpf larvae, as determined by *in situ* hybridization. Images shown are age-matched unlesioned controls for the lesioned animals shown in Fig. 1e (lateral view; rostral is left). n≥6 for each gene. Scale bars: 100 µm.

a-i) dpl, days post-lesion; expl. var., explained variance; FC, fold change; FDR, false discovery rate; padj, adjusted p value. The Source data are provided as a Source Data file.

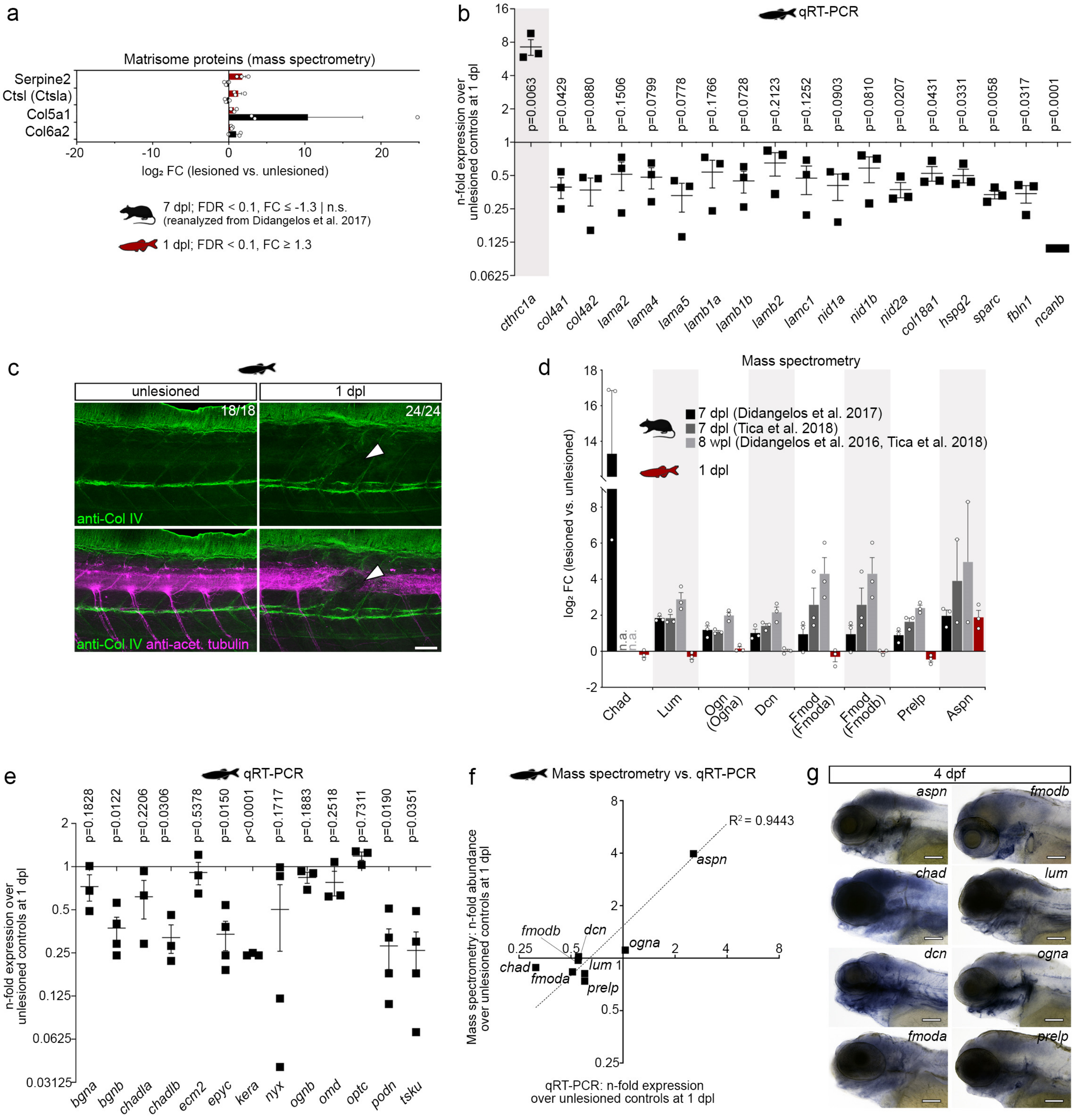

Supplementary Fig. 2 | Neurocan, basal lamina components, and small leucine-rich proteoglycans are not enriched in the zebrafish spinal lesion site.

a) Comparative proteomics analysis reveals differentially enriched matrisome proteins between rat (black) and zebrafish (red) after SCI. Shown are proteins that exhibit a high abundance (FDR < 0.1, FC ≥ 1.3) after SCI in zebrafish but a low abundance (FDR < 0.1, FC ≤ -1.3 | n.s.) in the rat spinal lesion site. Each data point represents one biological replicate. Data are means ± SEM.

b) Fold change expression of indicated genes in the zebrafish spinal lesion site at 1 dpl over unlesioned age-matched controls, as determined by qRT-PCR. Expression of indicated genes coding for neurocan b (Ncanb) and basal lamina constituents is not upregulated in the zebrafish spinal lesion site at 1 dpl. Note that *cthrc1a* served as a positive control for the detection of injury-induced expression of genes coding for ECM proteins [1]. Fold change values are presented in log

scale. Each data point represents one independent experiment. Data are means ± SEM. Paired two-tailed Student's t-test.

c) Immunolabeling of type IV collagen is not increased in the zebrafish spinal lesion site at 1 dpl (arrowheads). Neurites are labeled with immunofluorescence against acetylated tubulin (magenta). The number of specimens displaying the phenotype and the total number of experimental specimens is given. Images shown are maximum intensity projections of unlesioned trunk or lesion site (lateral view; rostral is left).

d) Comparative analysis of indicated proteomics datasets reveals differential enrichment of Chad, Lum, Ogna, Dcn, Fmoda, Fmodb, and Prelp proteins after SCI in rat (grey tones) and zebrafish (red). Note that Aspn is enriched after SCI both in rat and zebrafish. Each data point represents one independent experiment. Data are means ± SEM.

e) Fold change expression of indicated genes in the zebrafish spinal lesion site at 1 dpl over unlesioned age-matched controls, as determined by qRT-PCR. Expression of genes coding for indicated SLRP proteins is not upregulated at 1 dpl. Fold change values are presented in log scale. Each data point represents one biological replicate. Data are means ± SEM. Paired two-tailed Student's t-test.

f) Mean fold change values of indicated genes and proteins at 1 dpl over unlesioned age-matched controls as determined by qRT-PCR (Fig. 2b) and mass spectrometry-based quantitative proteomics (Supplementary Fig. 1c) are highly correlated ($R^2 = 0.9443$). Pearson correlation. Fold change values are presented in log scale.

g) Transcripts of indicated genes (blue) are detectable by *in situ* hybridization in whole-mount zebrafish larvae at 4 dpf. Images shown are lateral views of the heads of animals depicted in Fig. 2c.

a-g) Scale bars: 100 µm (**g**), 50 µm (**c**). dpf, days post-fertilization; dpl, days post-lesion; FC, fold change; FDR, false discovery rate; n.s., not significant; wpl, weeks post-lesion. The rat icon in panels (**a**) and (**d**) was created using BioRender. Source data are provided as a Source Data file.

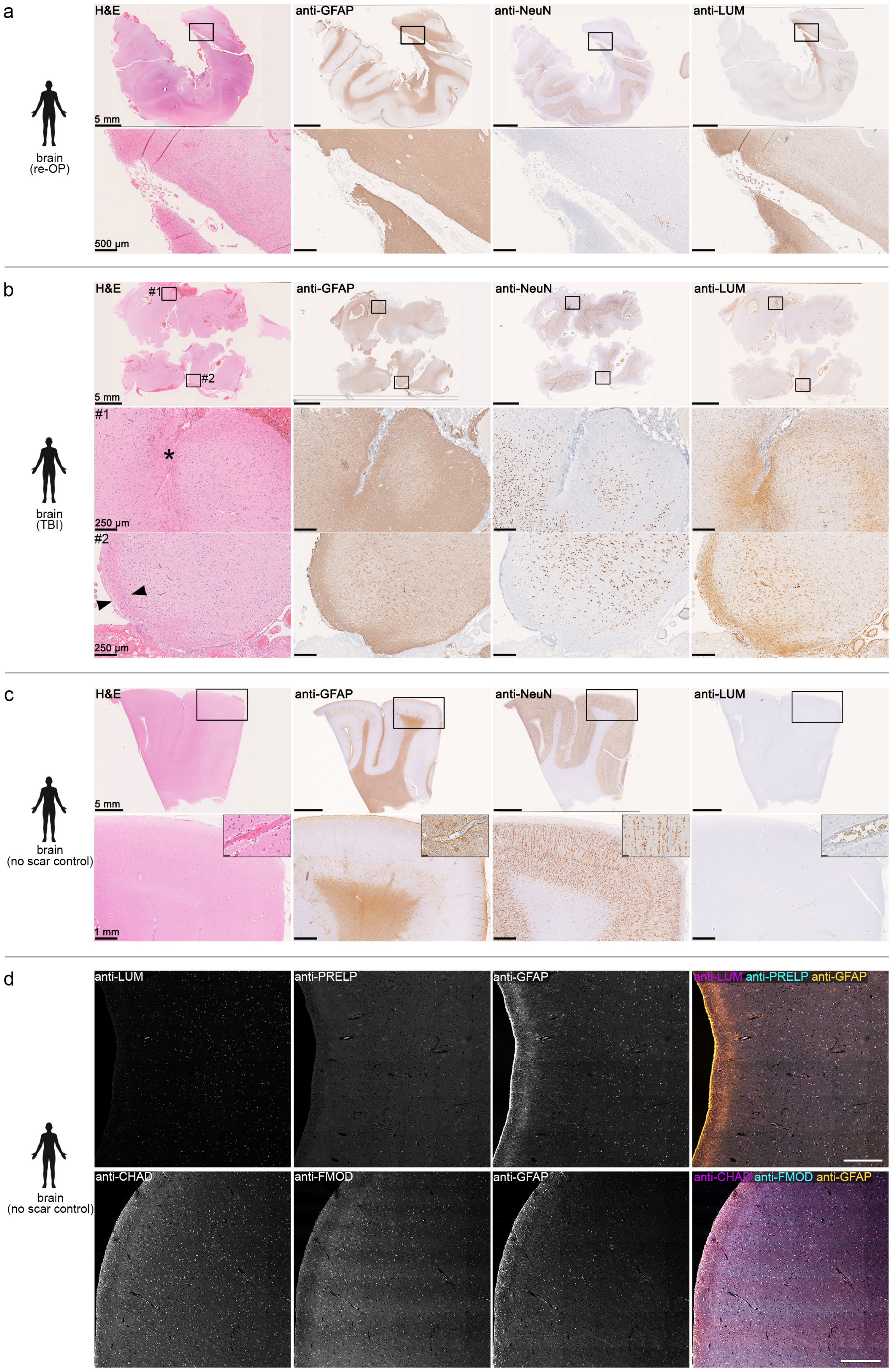

a
H&E
anti-GFAP
anti-NeuN
anti-LUM
5 mm
brain
(re-OP)
500 µm
b
#1
#2
brain
(TBI)
250 µm
c
brain
(no scar control)
1 mm
d
anti-PRELP
anti-LUM anti-PRELP anti-GFAP
anti-CHAD
anti-FMOD
anti-CHAD anti-FMOD anti-GFAP

Supplementary Fig. 3 | SLRPs are enriched in human brain lesions.

a-c) Immunohistochemical examination of human brain specimens. Hematoxylin and eosin (H&E) staining, and 3,3′-diaminobenzidine (DAB) staining of anti-GFAP, anti-NeuN, and anti-LUM antibodies on scarred brain tissue from patients with previous surgery (re-OP, **a**) or traumatic brain injury (TBI, **b**), and no scar control brain tissue (**c**). Areas of scarring were identified by H&E staining pattern and absence of immunoreactivity of the neuronal marker anti-NeuN. Anti-LUM immunoreactivity is increased in areas of scarring caused by previous surgery (**a**), contusion (arrowheads in **b**), or local hemorrhage (asterisk in **b**). Anti-LUM immunoreactivity is negligible in healthy human brain autopsy tissue with no signs of scarring (**c**). Shown are coronal sections. Size of scale bars is given in the figure. Six cases with scars following TBI or previous surgery, and six cases without scars were analyzed and showed similar results (Supplementary Table 1).

d) Anti-LUM, anti-PRELP, anti-CHAD, and anti-FMOD immunoreactivity is negligible in healthy human brain autopsy tissue with no signs of scarring. Images shown are immunofluorescence controls for data shown in Fig. 3. Six cases without scars were analyzed and showed similar results (Supplementary Table 1). Shown are coronal sections. Scale bars: 500 µm.

a-d) The human icon in panels (**a**), (**b**), (**c**), and (**d**) was created using BioRender.

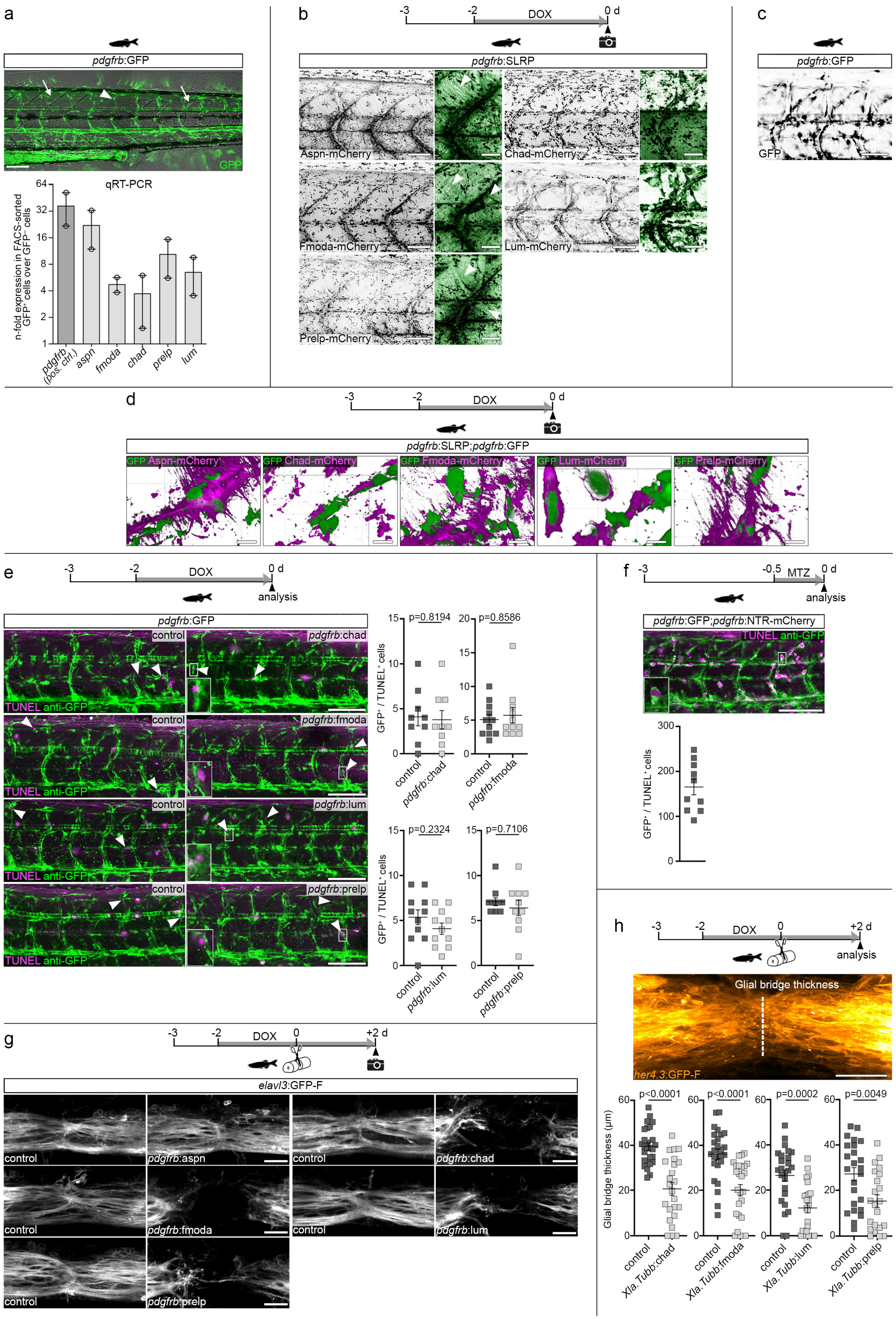

a
pdgfrb:GFP
GFP
qRT-PCR
n-fold expression in FACS-sorted GFP+ cells over GFP− cells
pdgfrb (pos. ctrl.)
aspn
fmoda
chad
prelp
lum
b
DOX
pdgfrb:SLRP
Aspn-mCherry
Chad-mCherry
Fmoda-mCherry
Lum-mCherry
Prelp-mCherry
c
pdgfrb:GFP
GFP
d
pdgfrb:SLRP;pdgfrb:GFP
GFP Aspn-mCherry
GFP Chad-mCherry
GFP Fmoda-mCherry
GFP Lum-mCherry
GFP Prelp-mCherry
e
analysis
pdgfrb:GFP
control
pdgfrb:chad
pdgfrb:fmoda
pdgfrb:lum
pdgfrb:prelp
TUNEL anti-GFP
p=0.8194
p=0.8586
p=0.2324
p=0.7106
GFP+ / TUNEL+ cells
f
MTZ
pdgfrb:GFP;pdgfrb:NTR-mCherry
TUNEL anti-GFP
g
elavl3:GFP-F
control
pdgfrb:aspn
h
Glial bridge thickness
her4.3:GFP-F
Glial bridge thickness (µm)
p<0.0001
p=0.0002
p=0.0049
Xla.Tubb:chad
Xla.Tubb:fmoda
Xla.Tubb:lum
Xla.Tubb:prelp

Supplementary Fig. 4 | *pdgfrb*$^+$ cell-specific targeting of SLRPs in zebrafish.

a) *pdgfrb*$^+$ myoseptal (arrows) and perivascular (arrowhead) cells (green) are a major source of endogenous SLRPs in uninjured larval zebrafish. Shown is the fold change expression of indicated genes in FACS-isolated GFP$^+$ cells over GFP$^-$ cells in trunk tissue of *pdgfrb*:GFP transgenic animals at 4 dpf, as determined by qRT-PCR. Note that *pdgfrb* served as positive control for the enrichment of *pdgfrb*$^+$ cells. Fold change values are presented in log scale. Each data point represents one independent biological replicate.

b) mCherry fluorescence (black) is robustly detected following induction of indicated *slrp-mCherry* fusions in *pdgfrb*$^+$ cells in *pdgfrb*:TetA;*TetRE*:SLRP-mCherry (short *pdgfrb*:SLRP) transgenic zebrafish. Note that the SLRP-mCherry fusions are secreted proteins and that the pattern of mCherry fluorescence resembles that of the *pdgfrb*$^+$ cell niche in *pdgfrb*:GFP transgenic animals, labeling mainly the myosepta and vasculature (compare (**a**) and (**c**)). Also note that the pattern of protein deposition varies among different SLRPs and that some exhibit mesh and fiber-like structures (arrowheads in magnified views; lookup table is shown). Similar results were obtained in n≥10 animals for each experimental condition.

c) *pdgfrb*$^+$ cells (black) in *pdgfrb*:GFP transgenic animals. Note that GFP is located in the cytoplasm. Similar results were obtained in n≥10 animals for each experimental condition.

d) *pdgfrb*$^+$ cell-specific induction of indicated *slrp-mCherry* fusions leads to pericellular labeling (magenta) of *pdgfrb*$^+$ cells (green) in *pdgfrb:SLRP*;*pdgfrb*:GFP transgenic animals. Note that the SLRP-mCherry fusions are secreted proteins and that the GFP protein is located in the cytoplasm. Similar results were obtained in n≥10 animals for each experimental condition.

e) Induction of the SLRPs *chad*, *fmoda*, *lum,* or *prelp* in *pdgfrb*:SLRP transgenic zebrafish does not increase the number of *pdgfrb*:GFP$^+$ cells (green) that undergo apoptosis (TUNEL$^+$, magenta; arrowheads). Insets show GFP$^+$/TUNEL$^+$ cells at higher magnification. Two-tailed Student's t-test (Chad, Lum, Prelp), two-tailed Mann-Whitney test (Fmoda). $n_{control}$=9, n_{Chad}=9; $n_{control}$=11, n_{Fmoda}=11; $n_{control}$=11, n_{Lum}=11; $n_{control}$=11, n_{Prelp}=10 animals.

f) Detection of a high number of GFP$^+$/TUNEL$^+$ (green/magenta) cells in *pdgfrb*:GFP;*pdgfrb*:NTR-mCherry transgenic zebrafish following metronidazole (MTZ) treatment, reveals efficient TUNEL probe penetration in unlesioned whole-mount preparations. Inset shows GFP$^+$/TUNEL$^+$ cells at higher magnification. n=10 animals.

g) *pdgfrb*$^+$ cell-specific induction of the SLRPs *chad*, *fmoda*, *lum*, or *prelp* but not *aspn* in *pdgfrb*:SLRP transgenic zebrafish reduces the thickness of the axonal bridge (white; analyzed in *elavl3*:GFP-F transgenics). Shown are example images of axonal bridges quantified in Fig. 5g.

h) *pdgfrb*$^+$ cell-specific induction of the SLRPs *chad*, *fmoda*, *lum*, or *prelp* in *pdgfrb*:SLRP transgenic zebrafish reduces the thickness of the glial bridge (lookup table is shown; analyzed in *her4.3*:GFP-F transgenics). Two-tailed Mann-Whitney test. $n_{control}$=27, n_{Chad}=24; $n_{control}$=26, n_{Fmoda}=26; $n_{control}$=25, n_{Lum}=24; $n_{control}$=24, n_{Prelp}=22 animals.

a-h) Images shown are maximum intensity projections or 3D reconstructions (**d**) of unlesioned trunk or lesion site (lateral view; rostral is left). Data are means ± SEM. Scale bars: 100 µm (**a**, **e**, **f**), 50 µm (**b**, **c**), 25 µm (**b** (magnified view), **g**, **h**)**,** 10 µm (**d**). d, days; DOX, doxycycline. Source data are provided as a Source Data file.

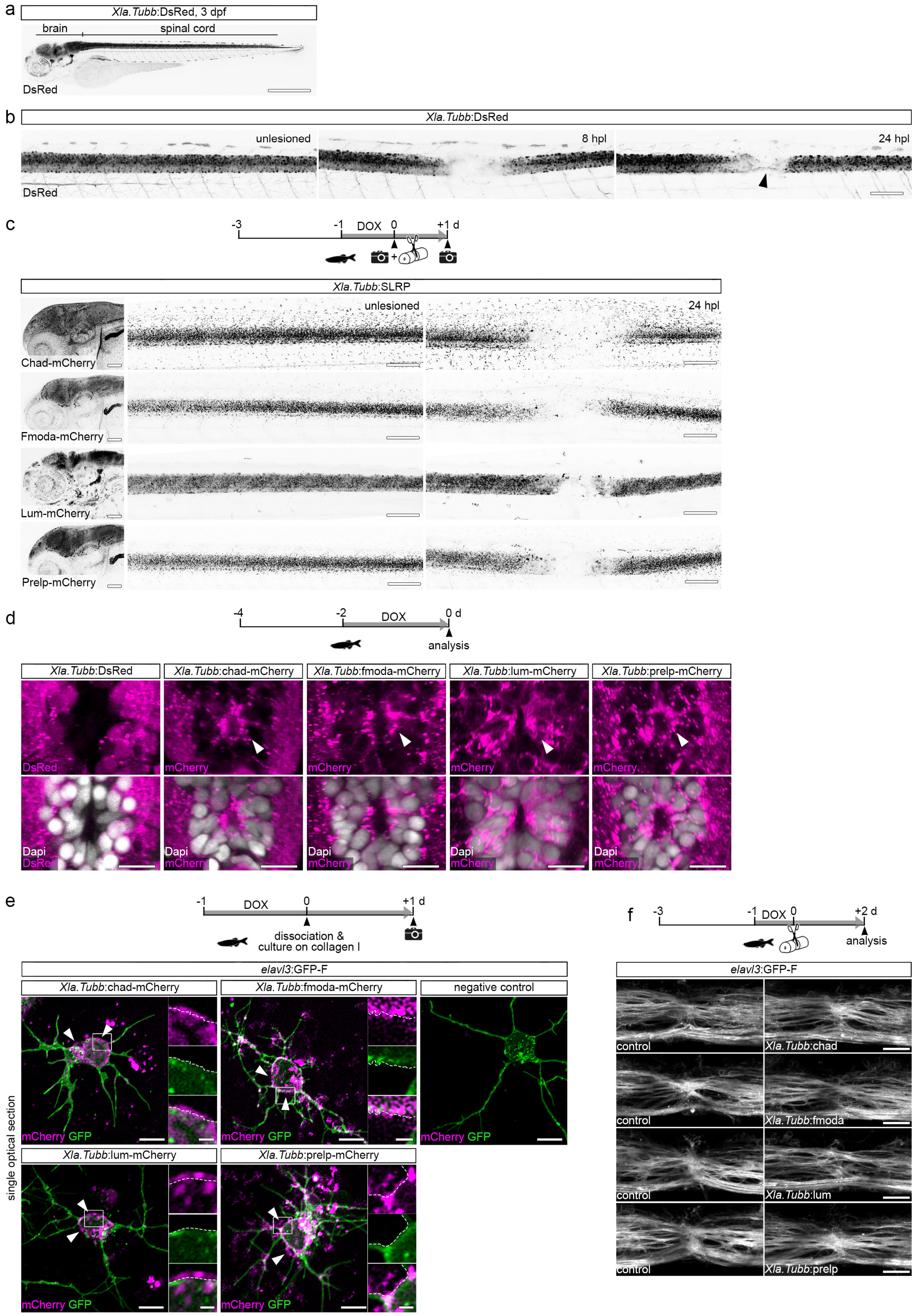
a
Xla.Tubb:DsRed, 3 dpf
brain
spinal cord
DsRed
b
Xla.Tubb:DsRed
unlesioned
8 hpl
24 hpl
DsRed
c
-3
-1
DOX
0
+1 d
Xla.Tubb:SLRP
unlesioned
24 hpl
Chad-mCherry
Fmoda-mCherry
Lum-mCherry
Prelp-mCherry
d
-4
-2
DOX
0 d
analysis
Xla.Tubb:DsRed
Xla.Tubb:chad-mCherry
Xla.Tubb:fmoda-mCherry
Xla.Tubb:lum-mCherry
Xla.Tubb:prelp-mCherry
DsRed
mCherry
Dapi
e
-1
DOX
0
+1 d
dissociation &
culture on collagen I
elavl3:GFP-F
Xla.Tubb:chad-mCherry
Xla.Tubb:fmoda-mCherry
negative control
Xla.Tubb:lum-mCherry
Xla.Tubb:prelp-mCherry
mCherry GFP
single optical section
f
-3
-1
DOX
0
+2 d
analysis
elavl3:GFP-F
control
Xla.Tubb:chad
Xla.Tubb:fmoda
Xla.Tubb:lum
Xla.Tubb:prelp

Supplementary Fig. 5 | Neuron-specific targeting of SLRPs in zebrafish.

a) The *Xla.Tubb* promoter drives transgene expression in all neurons of the brain and spinal cord [2]. Image shown is a maximum intensity projection of a *Xla.Tubb*:DsRed transgenic zebrafish larva at 3 dpf (rostral is left; dorsal is up). Fluorescence signal of the cytoplasmic DsRed protein is indicated in black.

b) Spinal cord transection leads to an acute neuronal loss, as indicated by the lack of DsRed fluorescence (black) in the lesion center of *Xla.Tubb*:DsRed transgenic animals at 8 hpl. Note that low fluorescence signal can be detected in the non-neuronal lesion core at 24 hpf due to the presence of cytoplasmic DsRed protein in regenerating axonal fibers (arrowhead). Images shown are maximum intensity projections of unlesioned trunk or lesion site (lateral view; rostral is left). Similar results were obtained in n≥10 animals.

c) mCherry fluorescence (black) is robustly detected and largely confined to the brain and spinal cord following induction of indicated *slrp-mCherry* fusions in neurons in *XlaTubb*:TetA;*TetRE*:SLRP-mCherry (short *Xla.Tubb*:SLRP) transgenic zebrafish. Note that the lesion core is largely devoid of fluorescence signal due to the absence of neuronal somata at 24 hpl (compare (**b**)). Also note the granular fluorescence pattern due to the extracellular localization of the SLRP proteins. Images shown are maximum intensity projections of the head, unlesioned trunk, or lesion site (lateral view; rostral is left). Similar results were obtained in n≥10 animals for each experimental condition.

d) *Xla.Tubb* promoter-driven expression of either *DsRed* (*Xla.Tubb*:DsRed) or indicated *slrp-mCherry* fusions (*Xla.Tubb*:SLRP) leads to different fluorescent labeling patterns (magenta) in the larval zebrafish spinal cord. Fluorescence signal is detected in the central canal and its ependymal lining in *Xla.Tubb*:SLRP (arrowheads) but not in *Xla.Tubb*:DsRed transgenic animals. Note that the DsRed protein is localized to the cytoplasm whereas SLRPs are secreted proteins. Also note that the *Xla.Tubb* promotor does not drive expression in ependymo-radial glia cells surrounding the central canal. Shown are magnified views of the images presented in Fig. 6c (transversal views of the spinal cord; dorsal is up). Similar results were obtained in n≥10 animals for each experimental condition.

e) Neuron-specific induction of indicated *slrp-mCherry* fusions leads to pericellular labeling (arrowheads; magenta) of primary neurons (green) prepared from dissociated *Xla.Tubb*:SLRP;*elavl3*:GFP-F transgenic animals. Note that the GFP protein is membrane-localized. Images shown are single optical sections at the level of the substrate-cell-interface. Similar results were obtained in n≥10 neurons for each experimental condition.

f) Neuron-specific induction of the SLRPs *chad*, *fmoda*, *lum,* or *prelp* in *Xla.Tubb*:SLRP transgenic zebrafish does not reduce the thickness of the axonal bridge (white; analyzed in *elavl3*:GFP-F transgenics) at 2 dpl. Shown are example images of axonal bridges quantified in Fig. 6d. Images shown are maximum intensity projections of the lesion site (lateral view; rostral is left).

a-f) Scale bars: 500 µm (**a**), 100 µm (**b**, **c**), 25 µm (**f**), 10 µm (**d**), 5 µm (**e**), 1 µm (magnified view in **e**). d; days; dpf, days post-fertilization; DOX, doxycycline; hpl, hours post-lesion.

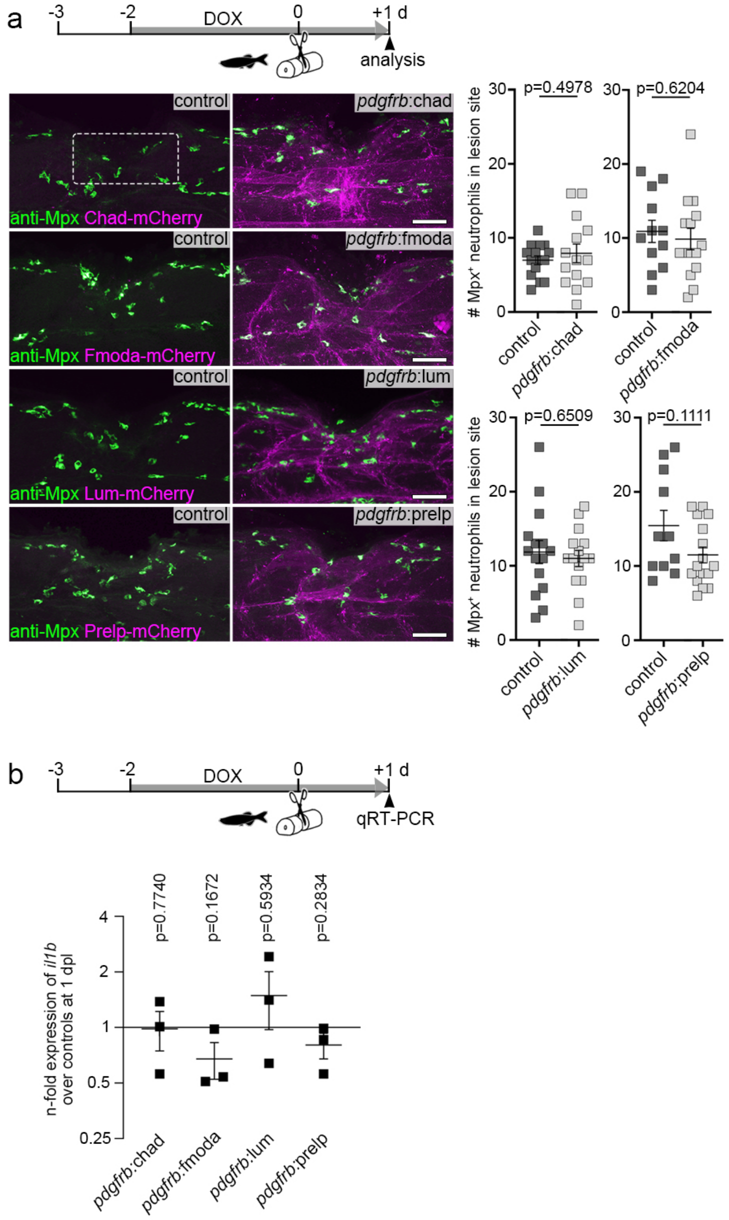

Supplementary Fig. 6 | Targeting SLRPs to the injury ECM does not prevent inflammation resolution.

a) Induction of *chad*, *fmoda*, *lum,* or *prelp* in *pdgfrb*:SLRP transgenic zebrafish does not lead to a higher number of Mpx^+ neutrophils in the lesion site at 1 dpl. Images shown are maximum intensity projections of the lesion site (lateral view; rostral is left). The dashed rectangle indicates the region of quantification. Each data point represents one animal. Two-tailed Student's t-test (Chad, Fmoda, Lum), two-tailed Mann-Whitney test (Prelp). $n_{control}$=15, n_{Chad}=14; $n_{control}$=12, n_{Fmoda}=15; $n_{control}$=15, n_{Lum}=15; $n_{control}$=11, n_{Prelp}=16 animals.

b) Fold change expression of *il1b* in the spinal lesion site of *pdgfrb*:SLRP transgenic zebrafish over controls at 1 dpl, as determined by qRT-PCR. Induction of *chad*, *fmoda*, *lum,* or *prelp* in *pdgfrb*:SLRP transgenics does not lead to increased expression levels of the proinflammatory cytokine *il1b*. Fold change values are presented in log scale. Each data point represents one independent biological replicate. Paired two-tailed Student's t-test.

a-b) Data are means ± SEM. Scale bars: 50 µm. d, days; dpl, days post-lesion; DOX, doxycycline. Source data are provided as a Source Data file.

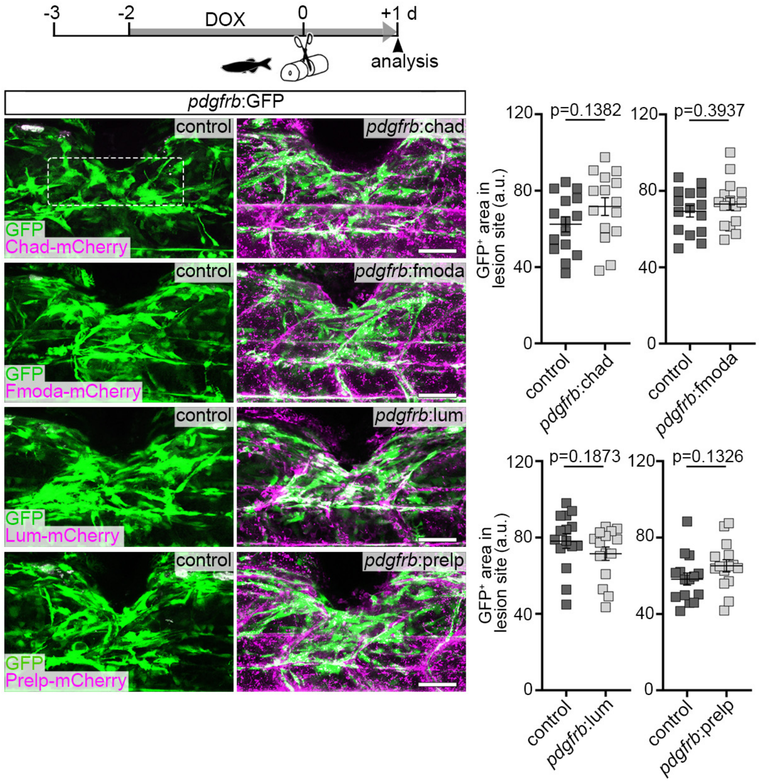

Supplementary Fig. 7 | *pdgfrb*⁺ cell-specific induction of SLRPs does not prevent recruitment of *pdgfrb*⁺ fibroblasts to the lesion site.

Induction of the SLRPs *chad*, *fmoda*, *lum,* or *prelp* in *pdgfrb*:SLRP transgenic zebrafish does not prevent the appearance of *pdgfrb*⁺ fibroblast cells (green) in the lesion site at 1 dpl. Images shown are maximum intensity projections of the lesion site (lateral view; rostral is left). The dashed rectangle indicates the region of quantification. Each data point represents one animal. Data are means ± SEM. Two-tailed Mann-Whitney test (Lum), Two-tailed Student's t-test (Chad, Fmoda, Prelp). $n_{control}$=15, n_{Chad}=15; $n_{control}$=15, n_{Fmoda}=15; $n_{control}$=15, n_{Lum}=15; $n_{control}$=15, n_{Prelp}=15 animals. Scale bars: 50 µm. a.u., arbitrary unit; d, days; DOX, doxycycline. Source data are provided as a Source Data file.

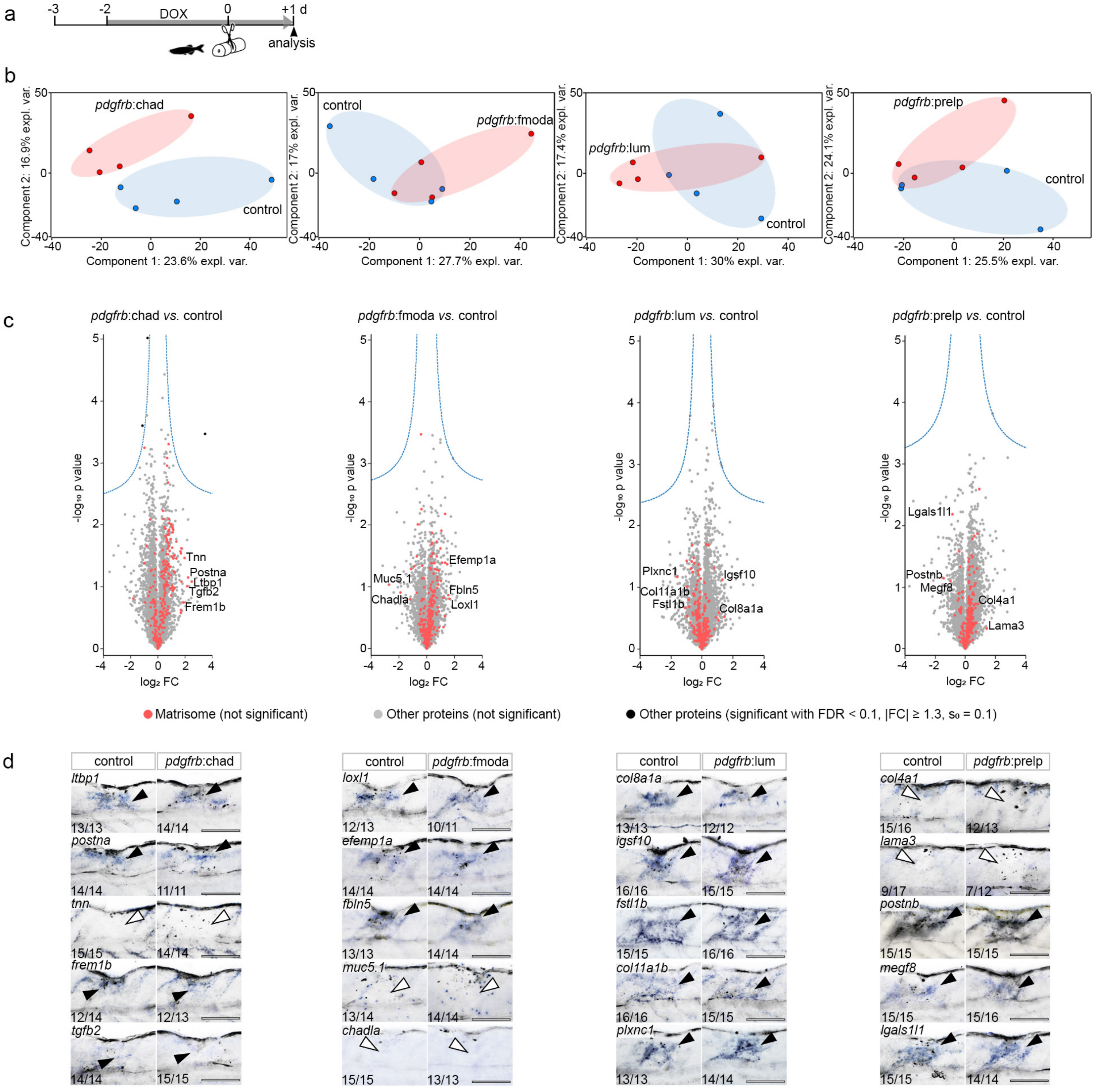

Supplementary Fig. 8 | Targeting SLRPs to the injury ECM does not alter its composition.

a) Timeline for experimental treatments shown in (**b**), (**c**), and (**d**).

b) Principle component analysis of mass spectrometry-based (MS) quantitative proteomics data from indicated experimental conditions, using quantitative values of all identified proteins across samples. Each data point represents one independent biological replicate.

c) Induction of the SLRPs *chad*, *fmoda*, *lum,* or *prelp* in *pdgfrb*:SLRP transgenic zebrafish does not lead to significant differences in the abundance of matrisome proteins at 1 dpl (red data points), as compared to controls. Shown are volcano plots of all quantified proteins for the given analyses with their $\log_2$-transformed ratios of the mean-centered abundances (FC, fold change) and $-\log_{10}$-transformed *P*-values (two-sided Student's t-test). Dashed lines indicate the threshold of a

permutation-based FDR correction for multiple hypotheses (FDR < 0.1, $s_0 = 0.1$) for identification of significantly altered abundances. Proteins with significantly altered abundance were further filtered by |FC| ≥ 1.3. Since the MS analysis cannot discriminate between endogenous and ectopic SLRP proteins, the respective manipulated protein has been excluded from the volcano plot. Indicated proteins were selected for verification by *in situ* hybridization (ISH; see (**d**)).

d) Induction of the SLRPs *chad*, *fmoda*, *lum*, or *prelp* in *pdgfrb*:SLRP transgenic zebrafish does not lead to major changes in the expression (blue) of indicated genes in the lesion site at 1 dpl, as determined by ISH. Transcript levels of five genes coding for matrisome proteins that showed the highest (yet non-significant) fold change in each of the four experimental conditions in (**c**) were evaluated. The number of animals displaying the phenotype and the total number of animals is given. Black arrowheads indicate ISH signal in the center of the lesion site, white arrowheads indicate absence of ISH signal. Images shown are brightfield recordings of the lesion site (lateral view; rostral is left). Scale bars: 100 µm.

a-d) d, days; DOX, doxycycline; expl. var., explained variance; FC, fold change; FDR, false discovery rate. Source data are provided as a Source Data file.

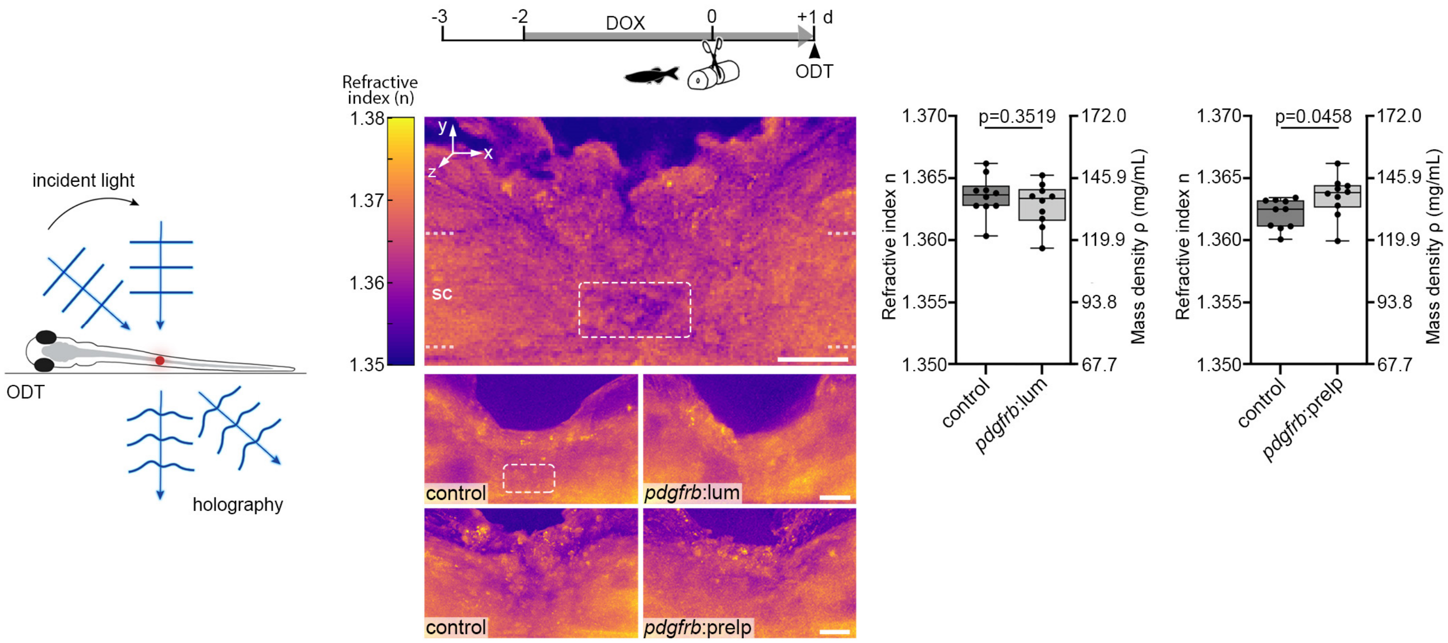

Supplementary Fig. 9 | Optical diffraction tomography analysis of the zebrafish spinal lesion site.

Optical diffraction tomography (ODT) analysis reveals the refractive index (n) of the lesion site in the indicated *pdgfrb*:SLRP transgenic zebrafish and their respective controls at 1 dpl. Images shown are sagittal optical slices through a refractive index tomogram of the zebrafish spinal lesion site (lateral view; rostral is left). The dashed lines indicate the location of the severed spinal cord, the dashed rectangles indicate the region of quantification. Each data point represents one animal. Box plots show the median, first, and third quartile. Whiskers indicate the minimum and maximum values. Two-tailed Student's t-test. $n_{control}$=10, n_{Lum}=10; $n_{control}$=10, n_{Prelp}=10 animals. Scale bar: 25 μm. d, days; DOX, doxycycline; sc, spinal cord. Source data are provided as a Source Data file.

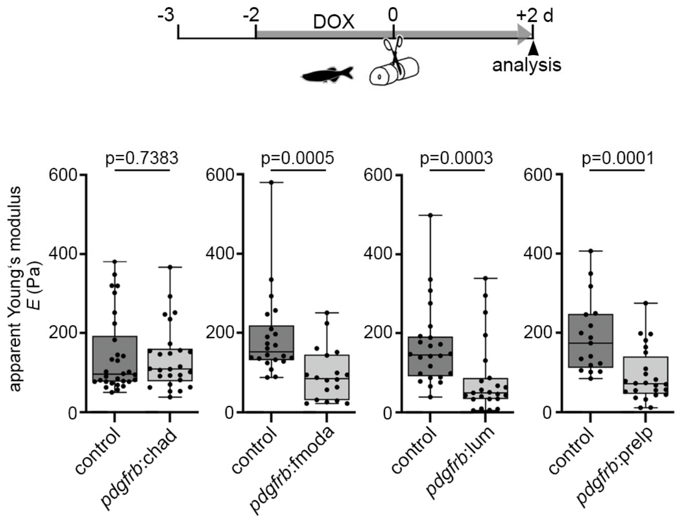

Supplementary Fig. 10 | Atomic force microscopy-based nanoindentation measurements of the zebrafish spinal lesion site.

Targeting Fmoda, Lum, or Prelp to the injury ECM in *pdgfrb*:SLRP transgenic zebrafish decreases the apparent Young's modulus (E) in the spinal lesion site at 2 dpl, as determined by atomic force microscopy-based nanoindentation measurements. Recorded force-indentation curves were fitted to the Hertz model (limitation of the indentation curve to 3 µm). Each data point represents one animal. Box plots show the median, first, and third quartile. Whiskers indicate the minimum and maximum values. Two-tailed Mann-Whitney test. $n_{control}$=30, n_{Chad}=26; $n_{control}$=22, n_{Fmoda}=18; $n_{control}$=24, n_{Lum}=23; $n_{control}$=17, n_{Prelp}=24 animals over three (Chad, Fmoda, Prelp) or four (Lum) independent experiments. DOX, doxycycline. Source data are provided as a Source Data file.

Supplementary Table 1 | Clinical data of human brain samples.

Case ID	Sex[1]	Age (years)	Onset (years)	Duration (years)	Side	Lobe	Diagnosis[2]	Cause[3]
B1	M	25	0	25	left	occipital	FCD 3D	TBI
B2	M	18	3	15	right	parietal	FCD 3D	TBI
B3	M	41	n/a	n/a	left	occipital	FCD 3D	TBI at 4 months of age
B4	F	6	0	6	left	temp occipital	FCD 1A	2nd surgery after 5 years
B5	M	10	2	8	left	temp occipital	FCD 1A	2nd surgery after 2 years
B6	F	2	0	2	left	temp occipital	FCD 1A	2nd surgery after 1 year
C1	M	10	0	10	right	occipital	FCD 1A	no scar control
C2	F	18	13	5	left	occipital	FCD 1A	no scar control
C3	M	27	18	9	left	frontal	mMCD	no scar control
C4	F	3	0	3	left	frontal	mMCD	no scar control
C5	F	49	-	-	-	frontal	autopsy	aorta aneurysm
C6	M	48	-	-	-	frontal	autopsy	heart failure

[1] M, male; F, female; [2] FCD, focal cortical dysplasia; mMCD, mild malformation of cortical development; [3] TBI, traumatic brain injury; abbreviations: n/a, not available.

Supplementary Table 2 | Clinical and neuropathological data of human spinal cord injury cases.

Case ID	Age at injury (years)	Sex[1]	Injury-death interval (days)	Tissue fixation post-mortem	Type of injury[2]	Level of injury[3]	AIS grade[4]	Diagnosis
BB2	60	M	111	5 days	C	C5	A	Hyperextension, avulsion flakes, traumatic disc
BB3	80	F	17	6 days	C/C	C6	B	Bilateral facet dislocation
BB4	76	M	34	13 hrs	C/C	C7	A	Three column fracture dislocation
BB5	82	M	9	12 hrs	C	C6	A	Bilateral facet dislocation
BB6	83	M	60	15 hrs	C/C	C4	D	Hyperextension, avulsion flakes, traumatic disc
BB9	95	M	15	1 day	C/C	C4	C	Hyperextension, avulsion flakes, traumatic disc

[1] M, male; F, female; [2] C/C, contusion/cyst; [3] C, cervical; number indicates vertebra; [4] AIS, American Spinal Injury Association Impairment Scale.

Supplementary Table 3 | Summary of results from immunofluorescence stainings on human spinal cord samples.

Case ID	Increased anti-CHAD immunoreactivity in E segment[1] as compared to R/C[2] segment?	Increased anti-FMOD immunoreactivity in E segment[1] as compared to R/C[2] segment?	Increased anti-LUM immunoreactivity in E segment[1] as compared to R/C[2] segment?	Increased anti-PRELP immunoreactivity in E segment[1] as compared to R/C[2] segment?
BB2	C	C	C	C
BB3	R ✓	R ✓	R ✓	R ✓
BB4	C	C ✓	C ✓	C ✓
BB5	R ✓	R ✓	R ✓	R ✓
BB6	C	C	C ✓	C
BB9	R	R ✓	R ✓	R ✓

[1] E segment, epicenter-containing segment; [2] R/C segment, segment located rostral or caudal to epicenter with little to no detectable pathology, ✓ indicates an observed increased immunoreactivity in E segments as compared to C or R segments of the same case.

Supplementary Note 1 | Longitudinal modulus and the mass density of binary mixtures.

The longitudinal modulus M' in dependence of wavelength λ, the refractive index n, mass density ρ and the Brillouin frequency shift ν_B in back scattering geometry is given by [3]

$$M' = \frac{\lambda^2 {\nu_\mathrm{B}}^2 \rho}{4\, n^2}.$$

Here we estimate the mass density of the sample as follows:

Based on the assumption of volume additivity, the total mass of solute (index 2) in a solvent (index 1) is given by

$$m = m_1 + m_2,$$

and the total volume

$$\begin{aligned} v &= v_1 + v_2, \\ &= v_1 + m_2\theta, \end{aligned}$$

where θ is the partial specific volume of the solute in mL/g. The mass of the solvent can now be expressed in terms of the total volume, density of the solvent, partial specific volume of the solute and the dry mass density $c_2 = m_2/v$

$$m_1 = \rho_1 v(1 - c_2\theta).$$

The mass density of the solution is accordingly given by

$$\rho = \frac{m}{v} = \rho_1 v(1 - c_2\theta) + c_2.$$

In order to connect the mass density with the refractive index of the solution, again, we assume volume additivity as

$$n = \varphi_1 n_1 + \varphi_2 n_2,$$

where φ_i is the volume fraction of the respective components, given by

$$\varphi_i = c_i/\rho_i.$$

Using $(1 - \varphi_2) = \varphi_1$, we arrive at

$$\frac{n - n_1}{c_2} = \frac{n_2 - n_1}{\rho_2} \approx \frac{\mathrm{d}n}{\mathrm{d}c} \equiv \alpha,$$

where α is the refractive index increment in mL/g, i.e. the slope of a $\mathrm{d}n(\mathrm{d}c)$ diagram. Finally, we arrive at an expression of the mass density in dependence of the refractive index as

$$\rho \approx \frac{n - n_1}{\alpha} + \rho_1 \left(1 - \theta \frac{n - n_1}{\alpha}\right).$$

This expression is not exact since $\theta = 1/\rho_2$ is not generally given.

Supplementary Note 2 | Uncertainty propagation for the longitudinal modulus.

In order to estimate the uncertainty associated with the longitudinal modulus we employ Gaussian propagation of uncertainty as

$$\Delta M' = \sqrt{\left(\frac{\partial M'}{\partial n}\Delta n\right)^2 + \left(\frac{\partial M'}{\partial \nu_B}\Delta \nu_B\right)^2 + \left(\frac{\partial M'}{\partial \alpha}\Delta \alpha\right)^2 + \left(\frac{\partial M'}{\partial \theta}\Delta \theta\right)^2},$$

where we account for the uncertainties in refractive index (n), Brillouin frequency shift (ν_B), refractive index increment (α) and partial specific volume (θ). The mean values and uncertainties of the refractive index and Brillouin frequency shift are obtained experimentally while the respective values of refractive index increment $(\alpha = (0.1919 \pm 0.0030)\ \mathrm{mL/g})$ and partial specific volume $(\theta = (0.743 \pm 0.010)\ \mathrm{mL/g})$ are theoretically calculated based on the zebrafish proteome ([4], Table 2 'Zebrafish', values are wavelength adjusted to $\lambda = 532\ \mathrm{nm}$). The explicit expression of the uncertainty of the longitudinal modulus is then

$$\Delta M' = \frac{\lambda^2 \nu_\mathrm{B}}{4n^3\alpha^2}\sqrt{(\xi_n \Delta n)^2 + \left(\xi_{\nu_\mathrm{B}}\Delta\nu_\mathrm{B}\right)^2 + (\xi_\alpha \Delta\alpha)^2 + (\xi_\theta \Delta\theta)^2},$$

with

$$\xi_n \equiv \alpha\nu_\mathrm{B}|n - 2n_\mathrm{fluid} + \rho_\mathrm{fluid}(2\alpha - n\theta + 2n_\mathrm{fluid}\theta)|,$$

$$\xi_{\nu_\mathrm{B}} \equiv 2n\alpha|n - n_\mathrm{fluid} - \rho_\mathrm{fluid}(\alpha + \theta(n_\mathrm{fluid} - n))|,$$

$$\xi_\alpha \equiv n\nu_\mathrm{B}|\theta\rho_\mathrm{fluid} - 1||n - n_\mathrm{fluid}|,$$

$$\xi_\theta \equiv n\alpha\nu_\mathrm{B}\rho_\mathrm{fluid}\,|n - n_\mathrm{fluid}|,$$

where the refractive index and density of water are $n_\mathrm{fluid} = 1.337$ and $\rho_\mathrm{fluid} = 0.9975\ \mathrm{g/mL}$, respectively. When evaluating the ratios of the uncertainty weights $\xi_n/\xi_{\nu_\mathrm{B}} \approx 0.42,\ \xi_\alpha/\xi_{\nu_\mathrm{B}} \approx 0.47,\ \xi_\theta/\xi_{\nu_\mathrm{B}} \approx 0.35$, we found them to be < 1 for all control and experimental measurement values of n and ν_B. Accordingly, if we consider the uncertainties of the longitudinal modulus associated with each parameter $\Delta M'_{\nu_\mathrm{B}} \approx 0.005\ \mathrm{GPa}$, $\Delta M'_n \approx 0.00018\ \mathrm{GPa}$, $\Delta M'_\alpha \approx 0.0013\ \mathrm{GPa}$ and $\Delta M'_\theta = 0.0032\ \mathrm{GPa}$, it becomes apparent that here the Brillouin frequency shift dominates the uncertainty content.

Supplementary References

1. Tsata V*, et al.* A switch in pdgfrb+ cell-derived ECM composition prevents inhibitory scarring and promotes axon regeneration in the zebrafish spinal cord. *Developmental cell* **56**, 509-524.e509 (2021).

2. Peri F, Nusslein-Volhard C. Live imaging of neuronal degradation by microglia reveals a role for v0-ATPase a1 in phagosomal fusion in vivo. *Cell* **133**, 916-927 (2008).

3. Boyd RW. *Nonlinear Optics, Third Edition*. Academic Press, Inc. (2008).

4. Zhao H, Brown PH, Schuck P. On the distribution of protein refractive index increments. *Biophysical journal* **100**, 2309-2317 (2011).
