# Supplementary material for: Small leucine-rich proteoglycans inhibit CNS regeneration by modifying the structural and mechanical properties of the lesion environment
Source: Nat Commun. 2023 Oct 26;14:6814. doi: 10.1038/s41467-023-42339-7 (PMC10603094; doi:10.1038/s41467-023-42339-7)
Supplement: Supplementary file 3 — Description of Additional Supplementary Files [file 41467_2023_42339_MOESM3_ESM.pdf]

Description of Additional Supplementary Files

Supplementary Data 1

Primers used for qRT-PCR and molecular cloning, and information on the source of in situ probe templates or primers used to generate in situ probe templates.

Supplementary Data 2

Plasmid map and sequence of construct used to the create Tg(*elavl3*:GFP-F)^{mps10} transgenic zebrafish line.

Supplementary Data 3

Plasmid map and sequence of construct used to create the Tg(*TetRE*:aspn-mCherry)^{mps11} transgenic zebrafish line.

Supplementary Data 4

Plasmid map and sequence of construct used to create the Tg(*TetRE*:chad-mCherry)^{mps12} transgenic zebrafish line.

Supplementary Data 5

Plasmid map and sequence of construct used to create the Tg(*TetRE*:fmoda-mCherry)^{mps13} transgenic zebrafish line.

Supplementary Data 6

Plasmid map and sequence of construct used to create the Tg(*TetRE*:fmoda-mCherry)^{mps14} transgenic zebrafish line.

Supplementary Data 7

Statistical tests, P-values, effect sizes and respective uncertainties, number of specimens.
